# Sol–Gel Synthesized CuFe_2_O_4_-Modified Biochar Derived from Tea Waste for Efficient Ni(II) Removal: Adsorption, Regeneration, and ANN Modeling

**DOI:** 10.3390/gels11080628

**Published:** 2025-08-10

**Authors:** Celal Duran, Sengul Tugba Ozeken, Serdal Seker, Duygu Ozdes

**Affiliations:** 1 Faculty of Sciences, Department of Chemistry, Karadeniz Technical University, Trabzon 61080, Türkiye; stozeken@gmail.com; 2 Graduate School of Education, Gumushane University, Gumushane 29100, Türkiye; serdalseker29@yahoo.com; 3 Gumushane Vocational School, Gumushane University, Gumushane 29100, Türkiye; duyguozdes@hotmail.com

**Keywords:** adsorption, ANN, biochar, CuFe_2_O_4_, nickel (II), sol-gel

## Abstract

In the present research, a novel magnetic adsorbent was developed via the sol–gel method by coating CuFe_2_O_4_ nanoparticles on biochar sourced from brewed tea waste. The synthesized adsorbent was utilized for the removal of Ni(II) ions from aqueous media. The adsorption efficiency of Ni(II) ions was assessed under crucial experimental conditions such as initial solution pH, contact time, adsorbent dosage, and initial Ni(II) concentration. The adsorbent exhibited rapid adsorption kinetics, achieving equilibrium in approximately 15 min, and maintained high efficiency across a wide pH range. Adsorption experiments were conducted for Ni(II) solutions at their natural pH (5.6) to minimize chemical usage and enhance process simplicity. An impressive maximum adsorption capacity of 232.6 mg g^−1^ was recorded, outperforming many previously reported adsorbents. Furthermore, desorption studies demonstrated nearly 100% recovery of Ni(II) ions using 1.0 M HCl solution, indicating excellent regeneration potential of the adsorbent. Additionally, the prediction performance of an artificial neural network (ANN) model was evaluated to predict Ni(II) removal efficiency based on experimental variables, showing strong agreement with experimental data. Isotherm and kinetic models were also applied to the data to estimate the adsorption mechanisms. These findings demonstrate the promise of CuFe_2_O_4_-modified tea waste biochar for sustainable water treatment applications.

## 1. Introduction

Heavy metals (HMs), dyes, and pharmaceuticals released from industrial activities contaminate water bodies as a result of the improper discharge of industrial wastewater. Nickel, widely used in the production processes of many products, such as batteries, electrical appliances, stainless steel, and alloys, is considered a significant environmental pollutant and is classified as a carcinogenic heavy metal [1,2,3,4]. Considering the importance of removing Ni (II) ions from wastewater, studies continue on the applicability, cost, and efficiency of new materials and techniques.

Membrane-based technologies, chemical methods, and methods based on electrical, photocatalytic, and adsorption processes have been applied to remove contaminated HMs before discharge [5]. Among these, adsorption-based methods stand out with numerous advantages such as offering easy operation, simple design, and rapid treatment processes by low energy consuming, eco-friendly, flexible and cost-efficient, and sometimes waste-reducing solutions for HM removal [4,5,6,7,8].

The selection of the adsorbent is crucial in adsorption processes due to the key role of interactions between the adsorbent and pollutant species as well as the significant properties such as quantity, accessibility, and chemical state of the active sites [9]. Various synthesized and natural materials have been tested for the removal of Ni(II) ions from aqueous media [9,10,11,12,13,14,15,16,17,18]. Among these, activated carbon is one of the most common adsorbent materials, but its high costs and the regeneration operations are the main drawbacks for industrial applications [19]. Therefore, research for new adsorbent materials performing high adsorption efficiencies continues.

Natural materials and wastes are among the interesting adsorbent materials because they align with the principles of green chemistry and offer versatile benefits in waste management, sustainability, and environmental protection. Biochar-based adsorbents, especially those derived from waste, have gained interest due to their adsorption capacities, porous structures, large surface areas, easy availability as a natural waste raw material, and low cost [20,21]. However, the adsorption efficiency of raw biochar is limited since the active sites on its porous surface are not co-energized, uniformly sized, and homogeneously distributed [22]. Additionally, high-temperature pyrolysis reduces the number of surface functional groups, thereby decreasing the active sites in biochar materials. Therefore, the metal adsorption capacity of pristine biochar should be improved by modification [5,21,23].

The adsorptive performances of various biochar materials [5,19,20,23,24,25] were investigated in Ni(II) ions’ retention. Incorporating metallic species into carbonaceous materials may enhance the adsorption capacity of raw biochar and enables overcoming the challenges of recovering the adsorbent from aqueous media. Magnetic properties were reported to enhance the metal ion adsorption behaviors of magnetic biochar materials due to various mechanisms such as electrostatic attractions, metal–π interactions, ion exchange, and complexation [24,26] in comparison to raw biochar materials. Moreover, inorganic oxides dispersed in a carbon matrix improve the surface functionalities, while metallic species with magnetic properties facilitate the recovery of adsorbent by simply applying a magnetic field [26,27].

As a significant material with chemical, photochemical, and thermal stability, electrical excellence, unique mechanical and magnetic properties, and low cost, CuFe_2_O_4_ attracts the attention of researchers among the other ferrite materials. On the other hand, CuFe_2_O_4_ nanoparticles are particularly notable due to their abundant surface functional groups (such as hydroxyl groups) and their suitability for surface modification. Compared to other transition metal ferrites like CoFe_2_O_4_ and NiFe_2_O_4_, CuFe_2_O_4_ generally exhibits lower toxicity and offers a more cost-effective synthesis route, primarily owing to the lower cost and higher natural abundance of copper precursors [28]. CuFe_2_O_4_ has been synthesized by several methods such as hydrothermal [29], co-precipitation [30], chemical vapor deposition [31], microwave combustion [32], high-energy ball-milling [33], electrochemical synthesis [34], and sol–gel [35]. Among various methods, the sol–gel method offers a significantly advantageous approach for the synthesis of new adsorbent materials. Simple design, low calcination temperature, and well-mixing and easy homogenization of the inexpensive precursors are the benefits of the sol–gel approach [36] to serve as a powerful method for the synthesis of ferrite materials [35].

There are many studies on the sol–gel synthesis of CuFe_2_O_4_ [35,36,37,38], differing in preparation procedures such as one or more of a solvent type, solution temperature, types and ratios of precursors, and calcination temperature in the literature. In the context of adsorption, CuFe_2_O_4_ has been reported to enhance the adsorption capacity of several adsorbents. At this point, the selection of the adsorbent and the method used to synthesize and incorporate CuFe_2_O_4_ becomes very important. In this study, as a powerful and useful method, the sol–gel method is preferred to synthesize CuFe_2_O_4_, while tea waste is utilized as a precursor to prepare a new and efficient magnetic biochar for the adsorptive removal of Ni(II) pollutant. Tea is widely used in beverage preparation and causes the formation of a large amount of solid waste after brewing. Tea waste (TW) has recently gained attention as a promising feedstock for biochar production, offering several advantages over traditional low-cost biomass sources. Its widespread availability and continuous generation at a global scale make it a sustainable and economically viable raw material. Chemically, TW consists of a complex mixture of organic compounds, including lignin, cellulose, hemicellulose, amino acids, proteins, polyphenols, and vitamins [39]. Diverse surface functional groups such as oxyl, phenolic hydroxyl, carboxylate, and aromatic carboxylate, generated through effective pyrolysis, can significantly enhance the surface reactivity and adsorption capacity of tea waste-derived biochar (TWB), making it particularly effective for removing heavy metal ions from aqueous media [40]. For this purpose, CuFe_2_O_4_ synthesized by the sol–gel method was introduced to TWB in the stage of carbonization to obtain an efficient magnetic tea waste biochar (MTWB) for removing Ni(II) from aqueous media.

Artificial intelligence (AI) is a powerful tool for solving complex problems that exceed the capabilities of traditional approaches. Among its subfields, machine learning stands out for enabling systems to learn and improve from data without requiring explicit programming. Within machine learning, artificial neural networks (ANNs) have gained significant recognition due to their high accuracy and adaptability in modeling complex, multivariate, and nonlinear relationships [41]. ANNs are increasingly used as efficient mathematical modeling tools in adsorption studies due to their ability to accurately predict complex and nonlinear relationships between the experimental results and variables [42]. Thanks to their high predictive accuracy, ANNs significantly reduce chemical consumption, shorten laboratory time, and minimize waste generation. Thus, they contribute to the development of more environmentally sustainable adsorption process designs.

In this study, the usability of biochar derived from brewed tea waste and modified with CuFe_2_O_4_ synthesized by sol–gel method was investigated as an adsorbent for the removal of Ni(II) ions. Existing adsorbents often exhibit limited pH applicability, slow kinetics, and poor regeneration. Here, a magnetic CuFe_2_O_4_/biochar composite was developed using brewed tea waste, combining improved structural properties with high adsorption efficiency. The work includes comprehensive adsorption–desorption analysis and employs an artificial neural network (ANN) model for performance prediction, offering a promising and sustainable approach for heavy metal removal from aqueous solutions.

## 2. Results and Discussion

### 2.1. Characterization

The morphological structures and elemental analyses of tea waste biochar (TWB) and CuFe_2_O_4_-modified brewed tea waste biochar (MTWB) were explored using scanning electron microscopy coupled with energy-dispersive X-ray spectroscopy (SEM-EDX). The specific surface area and porosity of MTWB used in the adsorption studies were determined at 77 K with N_2_ adsorption–desorption isotherms following the Brunauer–Emmett–Teller (BET) method. The crystal structures of TWB and MTWB were evaluated using X-ray diffraction (XRD) analysis, while the surface functional groups of both materials were investigated by utilizing a Fourier transform infrared spectrophotometer (FTIR).

**Figure 1:**
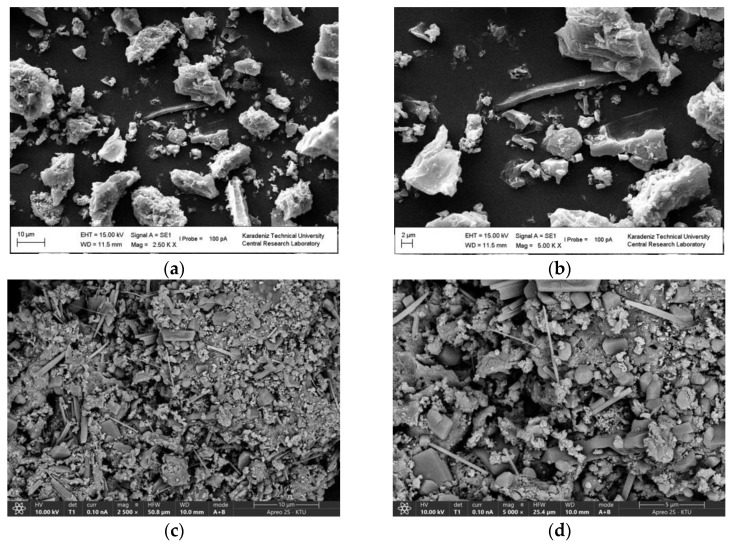
SEM images of TWB. (**a**) Low-magnification (2500×). (**b**) Higher magnification (5000×). SEM images of MTWB. (**c**) Low-magnification (2500×). (**d**) Higher magnification (5000×).

Surface morphologies were investigated by scanning electron microscopy (SEM) technique (Figure 1). SEM images with 2500× and 5000× magnifications represent the different morphologies of surfaces of tea waste biochar (TWB) and CuFe_2_O_4_-modified brewed tea waste biochar (MTWB). The SEM study clearly showed the morphological changes induced by the addition of CuFe_2_O_4_ during the calcination process. The surface of TWB, which is comparatively unstructured and smooth, exhibits limited morphological features and low complexity. However, SEM images of MTWB displayed a new and rich texture with densely dispersed particles, resulting from the formation of an inorganic phase throughout the material, as sol–gel synthesized CuFe_2_O_4_ was introduced into the calcination process. The emergence of new surface structures forming a more complex surface may lead to a larger surface area consisting of more vacant active sites for enhanced adsorption.

The elemental composition of TWB, as estimated by EDX analysis (Figures S1a–e and S2), demonstrated that the primary elements were carbon (C, 56.3%) and oxygen (O, 30.4%), along with nitrogen (N, 12.5%) and calcium (0.8%) (Table S1). However, after modification with CuFe_2_O_4_ via the sol–gel method, the elemental distribution changed significantly (Figures S3a–g and S4). The carbon and calcium contents decreased to 13.4% and 0.5%, respectively, while the oxygen content increased to 78.4% (Table S2). Additionally, iron (Fe, 4.8%) and copper (Cu, 2.5%) peaks were observed, confirming the successful incorporation of CuFe_2_O_4_ nanoparticles into the biochar matrix. The decrease in carbon content is attributed to the increased loading of metal oxide, which diminishes the relative carbon signal in EDX analysis. The increase in oxygen content may be attributed to the oxygen-rich nature of metal oxides (Fe–O and Cu–O bonds), as well as the possible formation of additional surface functional groups during the sol–gel process. A similar trend was reported by Kumar et al. [43], in the SEM-EDX analysis of nano magnetite (Fe_3_O_4_)-modified biochar derived from *Ascophyllum nodosum*.

The specific surface area, total pore volume, and micropore volume of MTWB were found to be 19.74 m^2^/g, 0.009023 cm^3^/g, and 0.008496 cm^3^/g, respectively, which is consistent with the previous literature [44].

**Figure 2:**
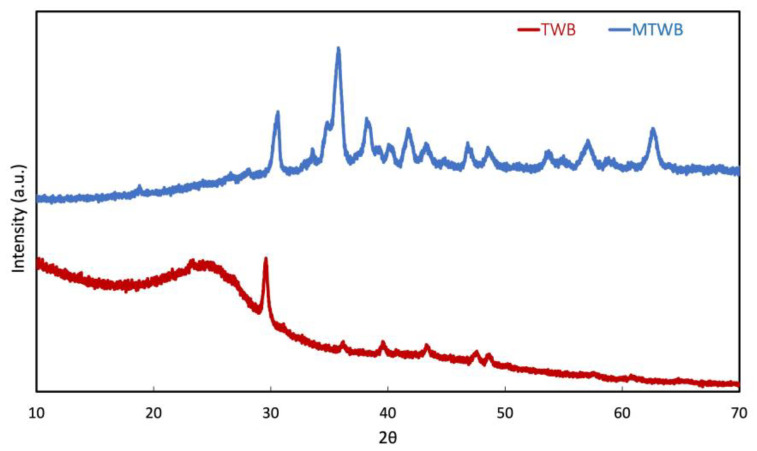
XRD patterns of TWB and MTWB.

XRD analysis was conducted to determine the phase composition and crystallographic structure of the synthesized materials. The results are presented in Figure 2. In the MTWB, several sharp and well-defined peaks were observed, indicating a high degree of crystallinity. The diffraction peaks located at 2θ = 18.3°, 30.2°, 35.6°, 43.3°, 53.7°, 57.3°, and 62.9° are attributed to the (111), (220), (311), (400), (422), (511), and (440) crystal planes of spinel-type CuFe_2_O_4_, as indexed by JCPDS card No. 77-0010 [45]. These results confirm the successful incorporation of CuFe_2_O_4_ into the biochar matrix during calcination. In contrast, the TWB sample exhibited a broad hump between 20° and 30°, characteristic of amorphous carbonaceous materials and associated with the (002) plane of disordered carbon structures. In addition, a distinct sharp peak at 2θ = 29.49°, indexed to the (104) plane of CaCO_3_, was detected. This peak matches well with JCPDS card No. 05-0586 and confirms the presence of calcite as a crystalline impurity [46]. The formation of this phase is attributed to the reaction between calcium present in the tea waste biochar and carbon dioxide released during the combustion of the organic matrix under calcination conditions, resulting in the formation of CaCO_3_. These results suggest that the integration of CuFe_2_O_4_ into the carbonaceous matrix improves the material’s crystallinity and alters its structural and chemical features, potentially enhancing its adsorption performance.

**Figure 3:**
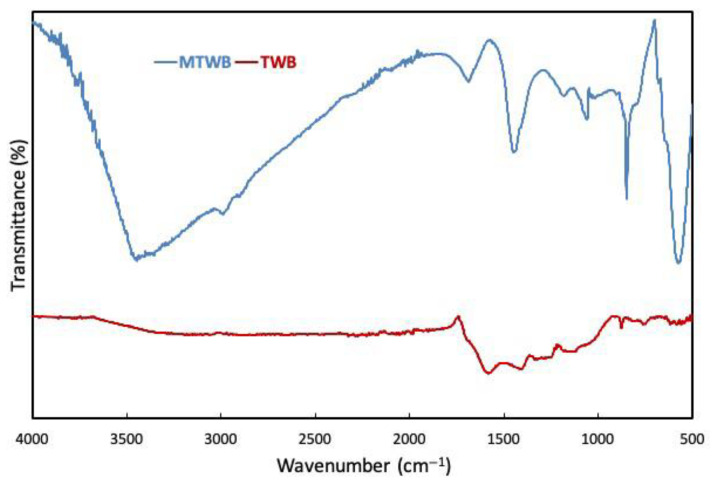
FTIR spectrums of TWB and MTWB.

Figure 3 represents the surface functional groups of TWB and MTWB determined by FTIR (Fourier transform infrared spectroscopy) technique (500–4000 cm^−1^). In the FTIR spectrum of TWB, O–H stretching vibrations of the hydroxyl (–OH) groups in alcohol and phenolic compounds were observed as a broad band between 3000 and 3600 cm^−1^. The stretching vibrations of C = O bonds in -COOH and ketones, and C = C bonds in aromatic rings were observed at 1581 cm^−1^ and 1410 cm^−1^, respectively. Moreover, the peaks observed at 875 cm^−1^ and 751 cm^−1^ may belong to the bending vibrations of C–H bonds observed in benzene derivatives. However, the FTIR spectrum of MTWB indicated the formation of new functional groups. The peaks observed at wavelengths of 574 cm^−1^ and 677 cm^−1^ were attributed to M-O structures (M: Fe and Cu) and small peaks at 870 cm^−1^, 970 cm^−1^, and 1457 cm^−1^ were ascribed to bending vibrations of M-OH structures. The broad band with a maximum value at 3435 cm^−1^ is ascribed to stretching vibrations of -OH groups. A peak located at 1640 cm^−1^ was assigned to the stretching vibrations of C = O bonds in carboxyl groups, and a strong peak at 1181 cm^−1^ was determined to belong to robust C-O group [47,48,49,50].

### 2.2. Impact of Parameters and Evaluation of Data

The initial and final concentrations of Ni(II) ions analyzed by FAAS were substituted into the following equation to calculate the adsorption capacity of MTWB.

(1) $$q_{e}=\frac{\left(C_{0}{-C}_{e}\right)\times V}{m_{s}}$$

Here, *q_e_* (mg g^−1^) is the adsorbed amount of Ni(II) ions per g of MTWB at equilibrium. *V* (mL) is the volume of the Ni(II) solution added to previously weighted amounts (*m_s_* (g)) of MTWB in each tube. *C*_0_ (mg L^−1^) and *C_e_* (mg L^−1^) are the initial and equilibrium concentrations of Ni(II) ions, respectively.

The influences of the initial pH of the Ni(II) solution, contact time of the adsorption process, dosage of CuFe_2_O_4_-modified tea waste biochar (MTWB), initial concentration of Ni(II) ions, and the presence of foreign salts in aqueous media on adsorption efficiency were investigated to optimize the conditions. Data were evaluated using kinetic models (pseudo-first order, pseudo-second order, and intraparticle diffusion), isotherm models (Langmuir, Freundlich, and Dubinin–Radushkevich), and artificial neural networks to obtain informative results and new predictions about the Ni(II) adsorbate–MTWB adsorbent system.

#### 2.2.1. Initial Solution pH

**Figure 4:**
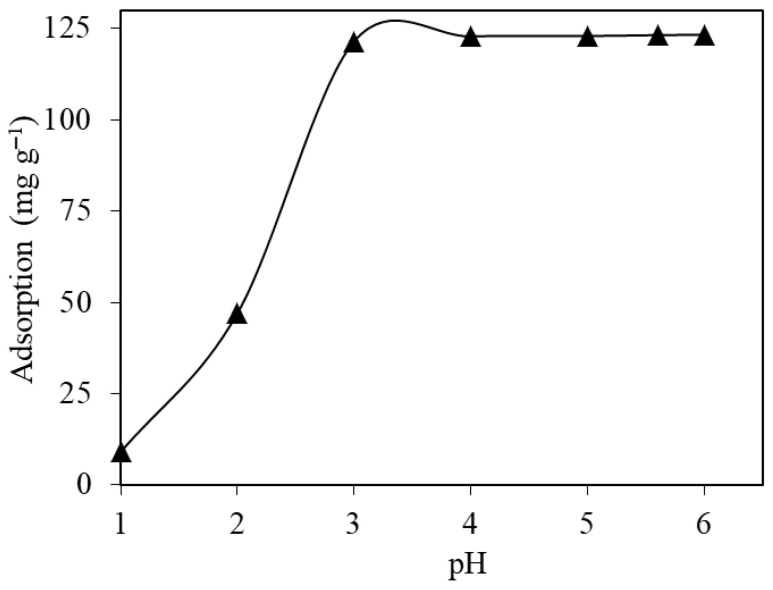
Effect of the initial solution pH on the uptake of Ni(II) ions (*C*_0_: 250 mg L^−1^; *V*: 10 mL; *m_s_*: 2.0 g L^−1^; *t*: 15 min).

The initial pH of the solution influences the charges of both the adsorbate and the surface of the adsorbent material. This effect can enhance or reduce the adsorptive performance due to possible changes in electrostatic attractions [51]. The pH values of the solutions containing 250 mg L^−1^ Ni(II) ions were adjusted to different values in the range of 1.0–6.0 using diluted HNO_3_ and NaOH solutions. An amount of 10 mL of pollutant solution was added to MTWB at a dosage of 2.0 g L^−1^, and the mixture was subjected to the adsorption process on a mechanical shaker for 15 min. The samples were centrifuged, and the Ni(II) concentration in the liquid phase was analyzed. The results were used to calculate the adsorption capacity of MTWB at each initial solution pH using Equation (1). The results, presented in Figure 4, revealed that the Ni(II) adsorption capacity of MTWB increased from 9.0 mg g^−1^ to 121.5 mg g^−1^ as the initial solution pH increased from 1.0 to 3.0. The adsorption capacity of MTWB became almost constant by then and reached 123.3 mg g^−1^ at pH 6.0. The Ni(II) adsorption capacity of MTWB, determined to be 123.2 mg g^−1^ at the natural initial pH value (5.6) of the Ni(II) solution, suggests that further studies can be performed at the natural pH and prevents the use of extra chemicals which is beneficial from an environmental perspective. The initial solution pH was optimized to be 5.6 (natural) for subsequent stages of the experimental studies.

#### 2.2.2. Contact Time

**Figure 5:**
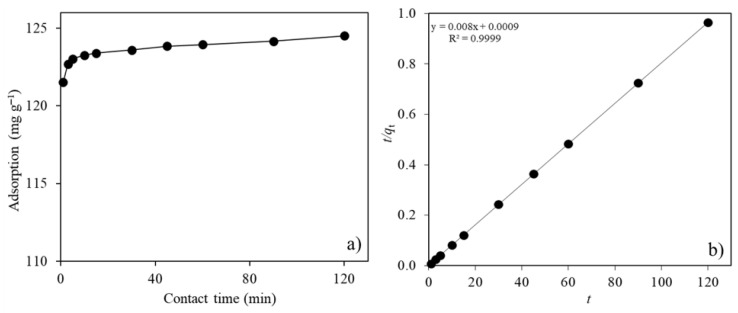
(**a**) Effect of contact time on the adsorption of Ni(II) ions (*C*_0_: 250 mg L^−1^; *V*: 10 mL; initial pH: 5.6 (natural); *m_s_*: 2.0 g L^−1^). (**b**) Pseudo-second order kinetic model.

To explore the effects of contact time, 10 mL of pollutant (250 mg L^−1^ Ni(II)) solution (natural pH: 5.6) was added to a 2.0 g L^−1^ MTWB suspension to prepare the test samples. The samples were subjected to an adsorption process for various contact times (1–120 min) to observe changes in the adsorption capacity of MTWB. Results (Figure 5a) showed that the Ni(II) adsorption capacity of MTWB increased to 123.4 mg g^−1^ within 15 min due to the rapid attachment of pollutant species to vacant available sites on MTWB. At longer contact times, no remarkable changes were observed in the adsorption capacity of MTWB. Further experiments were scheduled with 15 min of adsorption to ensure the sustainable use of energy resources.

Data were evaluated using kinetic models to investigate the dominance of different controlling mechanisms in the Ni(II) adsorption process. Pseudo-first order kinetic model (PFO), represented in linear form by Equation (2), is more accurate in the early stages of adsorption for an adsorbate–adsorbent system [52], while the pseudo-second order model (PSO), represented by Equation (3), describes the process over wider time range, including both initial and equilibrium stages [53].

(2) $$\ln(q_{e}-q_{t})=lnq_{e}-k_{1}t$$

(3) $$\frac{t}{q_{t}}=\frac{1}{k_{2}q_{e^{2}}}+\frac{t}{q_{e}}$$

where *q_e_* (mg g^−1^) and *q_t_* (mg g^−1^) are the adsorbed amounts of Ni(II) per g of MTWB at equilibrium and any given moment, respectively. *t* (min) represents the contact time. *k*_1_ (min^−1^) and *k*_2_ (g mg^−1^ min^−1^) are the rate constants of PFO and PSO models, respectively. The slope and the intercept of the ln(*q_e_* − *q_t_*) vs. *t* correspond to *k*_1_ and *q_e_* values in the PFO model, respectively. The slope and intercept of *t*/*q_t_* vs. *t* state *q_e_* and *k*_2_ values in the PSO model, respectively.

Moreover, the intraparticle diffusion model (IPD) [54] reinforces the explanation of the process by defining adsorption in three stages for deepening. Here, the adsorbate species first diffuse to the film layer (film diffusion), then penetrate the interface, diffuse across it to reach the pores (pore diffusion), and finally get rapidly adsorbed onto the pores. The reaction rate is generally controlled by the slower one of the first or second stages. The IPD model is written and presented in linear form as shown in Equation (4):

(4) $$q_{t}=k_{id}t^{1/2}+C$$

where *q_t_* (mg g^−1^) is the instantaneous amount of Ni(II) retained by 1 g of MTWB, *k_id_* (g mg^−1^ min^−1/2^) is the rate constant, and *t* (min) is the contact time. Here, *C* is a constant that marks the thickness of the boundary layer. *q_t_* vs. *t*^1/2^ plot figures out *k_id_* and C parameters by the slope and intercept, respectively, thereby describing a process consisting of three stages, if multilinear, as explained above. The intraparticle (pore) diffusion stage limits the rate of adsorption if *q_t_* vs. *t*^1/2^ plot passes through the origin [55].

The time dependency of Ni(II) adsorption was investigated through the rate constants of the PFO, PSO, and IPD models. According to Table 1, the calculated Ni(II) adsorption capacity (*q_e cal_*) of MTWB was 1.81 mg g^−1^ for the PFO model and 125 mg g^−1^ for the PSO model, while correlation coefficients (*R*^2^) were 0.8818 and 0.9999, respectively. Here, the *q_e cal_* value of the PSO model is compatible with the experimental amount of adsorbed Ni(II) (*q_e exp_*), which was determined to be 124.5 mg g^−1^. The correlation coefficient was higher than 0.999 and very close to linear in the PSO model (Figure 5b). In contrast the PFO model was insufficient for explaining the mechanisms in Ni(II) adsorption to MTWB in this study. Furthermore, since the rate constant calculated for pore diffusion (intraparticle diffusion) (*k*_id,2_) is smaller than the one calculated for film diffusion (*k*_id,1_), Ni(II) adsorption on CuFe_2_O_4_-modified tea waste biochar (MTWB) seems to be appearing under the control of pore diffusion. However, the nonzero *C* value reveals intricate mechanisms under the joint control of film and pore diffusion [56].

#### 2.2.3. Adsorbent Dosage

The adsorbent dosage influences the dynamics and effectiveness of the adsorbate–adsorbent system by determining the total number of active sites. Increasing the adsorbent dosage can improve the adsorbate’s removal efficiency due to a larger surface area containing numerous accessible active sites available for adsorption [57]. However, beyond the optimum dosage, a contrarily decreasing trend in adsorption capacity is often observed due to possible aggregations resulting in a reduced number of vacant sites. Furthermore, sub-dosed applications can result in inaccurate calculations due to a small amount of adsorbed solute, while overdoses can alter the chemistry of the solution. In some cases, this alteration may manifest as a pH change (or an ionic strength) that indirectly influences the effectiveness of adsorption. For this reason, it is essential to optimize the adsorbent dosage to maximize efficiency and minimize cost and waste [51,58].

**Figure 6:**
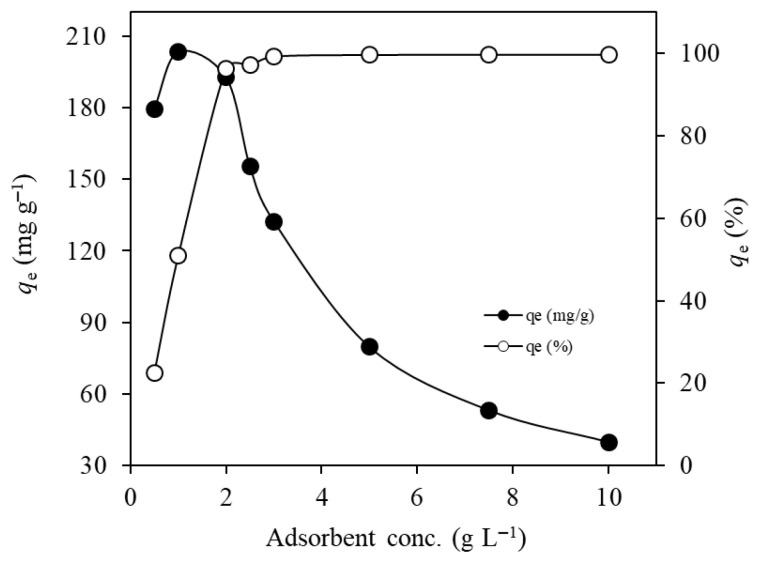
Effect of adsorbent amount on the adsorption of Ni(II) ions (*C*_0_: 400 mg L^−1^; *V*: 10 mL; initial pH: 5.6 (natural); *t*: 15 min).

In total, 10 mL of Ni(II) solution (400 mg L^−1^ initial concentration and natural initial pH of 5.6) was added to MTWB in varying dosages between 0.50 and 10.0 g L^−1^ and subjected to adsorption for 15 min (Figure 6).

According to Figure 6, the Ni(II) adsorption capacity reached its maximum value (203.4 mg g^−1^) at an MTWB dosage of 1.0 g L^−1^ with an adsorption percentage of 50.9%. MTWB dosages increasing beyond 1.0 g L^−1^ resulted in a gradually decreasing trend in adsorption capacity ultimately dropped to 39.9 mg g^−1^ at the maximum dosage (10.0 g L^−1^) due to possible agglomeration. Conversely, the percentage of adsorption dramatically increased to 96.4% by doubling the dosage (2.0 g L^−1^), and then slightly increased in the same direction and finally reached a value of 99.7% for the highest dosage (10.0 g L^−1^) due to high amounts of adsorbent material consisting of more numbers of active sites available for the adsorption of Ni(II) at a constant concentration [57].

#### 2.2.4. Ni(II) Concentration and Adsorption Isotherms

The initial concentration of the adsorbate is one of the foremost variables that influence the adsorption process by generating a mass transfer gradient at the interface between the bulk solution and the adsorbent′s surface. Thus, adsorption efficiency tends to be higher at low initial adsorbate concentrations since most of the active sites remain unconquered. However, the vacant active sites become closer to saturation as the initial adsorbate concentration increases and usually results in a higher adsorption capacity due to an enhanced concentration gradient that ensures expedited diffusion at the interface. Still, the comprehensive adsorption efficiency diminishes or forms a plateau since the adsorptive sites become saturated if the number of active sites on the surface is limited, even though the initial concentration of the solute is very high [59]. Thus, the adsorbate concentration needs to be optimized to prevent underscored adsorption capacity.

**Figure 7:**
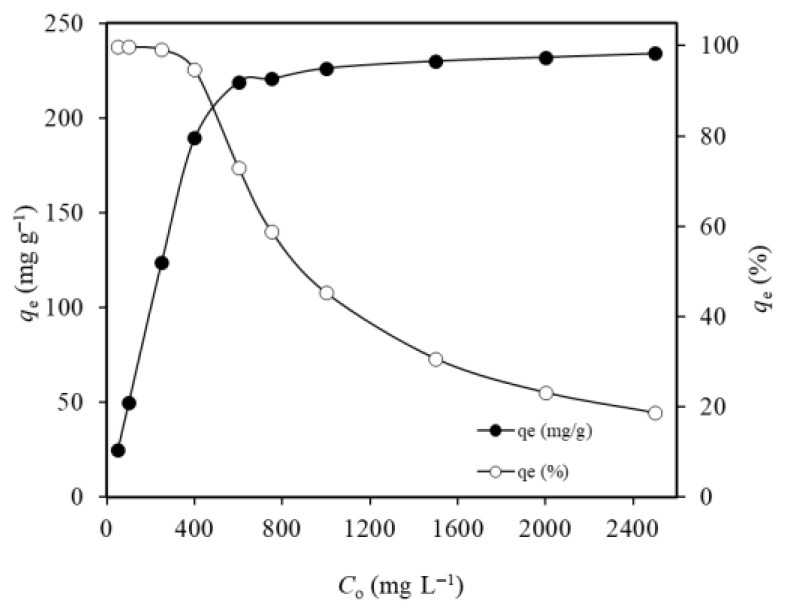
Effect of initial Ni(II) concentration on its uptake (*V:* 10 mL; *m_s_*: 2.0 g L^−1^; *t*: 15 min).

Test solutions, containing various concentrations of Ni(II) ions at a natural pH value of 5.6, were processed with 2.0 g L^−1^ MTWB for 15 min for adsorption. Results (Figure 7) showed that as the initial concentration of Ni(II) increased from 0.05 g L^−1^ to 2.50 g L^−1^, the adsorption capacity of MTWB increased from 25.0 mg g^−1^ to 234.0 mg g^−1^, indicating the attachment of numerous Ni(II) ions to the available active sites of MTWB. However, the percentage of adsorption tended to decrease as the Ni(II) concentration increased. The percentage of adsorption exceeded 99.0% in the experimental studies where the initial Ni(II) concentration was equal to or less than 0.25 g L^−1^ due to the abundance of available sites for the attachment of Ni(II) ions. The percentage of adsorption decreased from 94.7% to 18.7% as the initial concentration of Ni(II) increased from 0.4 g L^−1^ to 2.50 g L^−1^. This result was attributed to the saturation of a fixed number of active sites relative to a high number of Ni(II) ions at elevated initial pollutant concentrations. Hence, the excess Ni(II) ions could not attach to occupied sites, and the ratio of concentrations of adsorbed Ni(II) ions to unadsorbed Ni(II) ions decreased [60,61]. Jock et al. (2021) observed a similar trend in the adsorption of Ni(II) on Nigerian bentonite [62].

Mathematical isotherm models are used to gain deeper insights into precisely characterizing the behavior of the adsorbate–adsorbent system at equilibrium and to take the advantage of establishing a relationship between the concentration of the adsorbate in the solution phase and its amount adsorbed on the adsorbent at a constant temperature. The Langmuir, Freundlich, and Dubinin–Radushkevich (D-R) isotherms are often employed among readily accessible models thanks to their applicability to various adsorbents with different surfaces and their differing conceptual foundations.

The conceptual basis of the Langmuir model is based on the notion that adsorption occurs across the entire surface consisting of equally energized sites and provides the best model for the uniform adsorption of the adsorbate as a single layer. As each available site of the adsorbent is occupied by an adsorbate molecule, no additional adsorption occurs, and no interactions take place between the adjacent species in this model [61,63]. The Langmuir model, presented by Equation (5) in linear form, is as follows:

(5) $$\frac{C_{e}}{q_{e}}\;=\;\frac{C_{e}}{q_{max}}+\frac{1}{{bq}_{max}}$$

And the suitability of the adsorption can be assessed by an additional equation (Equation (6)) as follows:

(6) $$R_{L}\;=\;\frac{1}{{1+bC}_{0}}$$

where *q_e_* (mg g^−1^) is the unit Ni(II) adsorption at equilibrium, while *q_max_* (mg g^−1^) denotes the maximum monolayer adsorption capacity of MTWB. *C_e_* (mg L^−1^) is the concentration of the unadsorbed Ni(II) ions at equilibrium, *C*_0_ (mg L^−1^) is the initial concentration of Ni(II) ions, *b* (L mg^−1^) is a constant related to the enthalpy of adsorption, and *R_L_* (dimensionless) is the separation factor. The *C_e/_q_e_* vs. *C_e_* plot is used to obtain *b* and *q_max_* with the intercept and slope, respectively, and to assess the suitability of the model through the linearity. *R_L_* (a dimensionless parameter) serves information about the compatibility of adsorption through four possible evaluations: favorable (0< *R_L_* < 1), unfavorable (*R_L_* > 1), linear (*R_L_* = 1), or irreversible *(R_L_* = 0) [61].

The Freundlich isotherm provides an empirical model that describes multilayer adsorption of adsorbate species with logarithmically distributed binding energies over a non-ideal and heterogeneous adsorbent surface [64]. The model differs from the Langmuir model in its applicability to the adsorption behaviors of a non-uniformly energized surface with unlimited saturation. The linearized equation of the Freundlich model is presented in Equation (7),

(7) $$\ln q_{e}=\ln K_{f}+\frac{1}{n}C_{e}$$

where *q_e_* (mg g^−1^) is the Ni(II) adsorption capacity at equilibrium, *K_f_* (mg g^−1^) is the Freundlich constant related to the capacity of adsorption, *n* is the adsorption intensity, and *C_e_* (mg L^−1^) is the equilibrium concentration of unadsorbed Ni(II) ions.

The slope and intercept in the plot of *ln C_e_* vs. *ln q_e_* state the *1*/*n* value and *K_f_*, respectively. The current process is considered to be favorable for the adsorbate–adsorbent system if the *n* corresponds to a value in the 0–1 range [61].

The Dubinin–Radushkevich (D–R) model serves as an extensive framework for evaluating the mechanisms of adsorption, especially on irregular surfaces, and differs from the Langmuir and Freundlich models by introducing the concept of adsorption potential to characterize adsorption as physical or chemical [65]. The linearized equation of the D-R model is presented in Equation (8).

(8) $$\ln q_{e}=\ln q_{m}-\beta \varepsilon^{2}$$

where *q_e_* (mg g^−1^) is the Ni(II) adsorption capacity of MTWB at equilibrium, *β* (mol^2^ kJ^−2^) is the activity coefficient related to the energy of Ni(II) adsorption, *q_m_* (mg g^−1^) is the monolayer adsorption capacity of Ni(II) ions, and *ԑ* is the Polanyi potential. The slope and intercept of the linear ln *q_e_* versus *ε*^2^ plot denote *β* and *q_m_*, respectively. Equation (9) is used to calculate the mean adsorption energy. Hence, an adsorption process is evaluated to involve chemical (*E* > 16 kJ mol^−1^), physical (*E* < 8 kJ mol^−1^), or ion exchange-based (8 kJ mol^−1^ < *E* < 16 kJ mol^−1^) mechanisms [66].

(9) $$E=\frac{1}{{\left(-2\beta \right)}^{1/2}}$$

**Figure 8:**
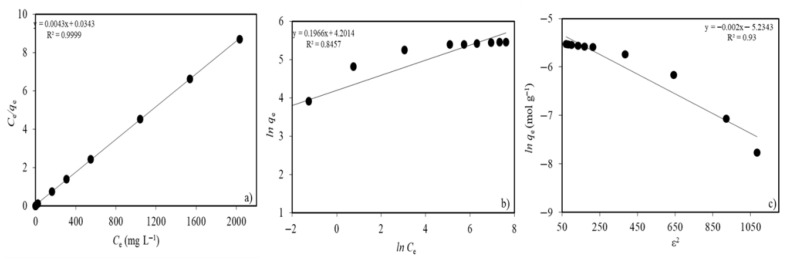
(**a**) Langmuir isotherm model; (**b**) Freundlich isotherm model; (**c**) Dubinin–Radushkevich isotherm model.

Figure 8a–c were plotted to represent the evaluated results of the Langmuir, Freundlich, and Dubinin–Radushkevich isotherm models in the adsorption of Ni(II) ions on MTWB, respectively. Moreover, the isotherm parameters were tabulated (Table 2).

According to Table 2, the maximum Ni(II) adsorption capacity of MTWB was determined to be 232.6 mg g^−1^ using the Langmuir isotherm model. To further assess the impact of CuFe_2_O_4_ modification on the adsorption capacity, adsorption isotherms were also evaluated for raw tea waste biochar using Ni(II) ion concentrations in the range of 50–1000 mg L^−1^. According to the Langmuir isotherm model, the adsorption capacity of TWB was obtained as 16.4 mg g^−1^. The enhanced performance of MTWB supports the modifying role of copper ferrite additives in increasing adsorption efficiency. For the adsorption of Ni(II) ions onto MTWB, when the correlation coefficients were compared, the Langmuir model (*R*^2^ = 0.9999) provided a better fit and a more linear definition than the Freundlich model (0.8457) for the Ni(II) adsorbate–MTWB adsorbent system. This result appears consistent with monolayer adsorption and suggests the presence of strong site-specific binding of Ni(II) on MTWB’s active sites. The mean adsorption energy, determined to be 15.8 kJ mol^−1^, supports the dominance of ion exchange-based mechanisms in the process. The *n* value (5.1) and *R_L_* values, falling within the 0–1 range, declared the suitability of Ni(II) adsorption on MTWB under the current conditions.

The key adsorption parameters of Ni(II) adsorption on MTWB—including the Langmuir maximum adsorption capacity, optimum pH, adsorbent dosage, and contact time— were compared with those of adsorbent materials reported in the literature [9,10,11,12,14,19,21,24,25,67]. The MTWB adsorbent can serve as an efficient material for industrial wastewater treatment, as its Ni(II) adsorption capacity is higher than that of many adsorbent materials, including various biochar-based adsorbents and activated carbons (Table 3). In addition to its remarkably high adsorption capacity (232.6 mg g^−1^), MTWB displayed exceptionally rapid adsorption kinetics, reaching equilibrium in approximately 15 min. The material also maintained a consistently high Ni(II) removal efficiency over a broad pH range, indicating robust performance under varying environmental conditions. Conducting adsorption experiments at the natural pH of the solution (5.6) minimized the need for chemical usage, thereby enhancing operational simplicity and environmental compatibility.

#### 2.2.5. Desorption

**Figure 9:**
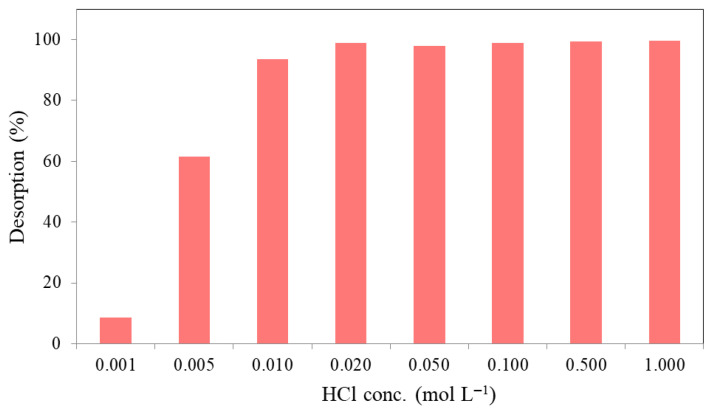
Desorption of Ni(II) ions (*C*_0_: 250 mg L^−1^, *V*: 10 mL; initial pH: 5.6, *m_s_*: 2.0 g L^−1^, *t*: 15 min).

A total of 10 mL of aqueous solution containing 250 mg L^−1^ Ni(II) ions at the natural pH value of 5.6 was processed for adsorption with 2.0 g L^−1^ MTWB for 15 min in each test. Each suspension was centrifuged to separate the phases at the end of the process, and the solid phase was treated with variously concentrated (0.001–1.0 mol L^−1^) HCl solutions in batch processes to ensure 15 min of desorption of Ni(II) from the MTWB. Results, presented in Figure 9, showed that as the concentration of HCl solution increased from 0.001 mol L^−1^ to 0.01 mol L^−1^, Ni(II) desorption percentage increased from 8.5% to 93.4%, and for higher concentrations (>0.01 mol L^−1^) of HCl, the percentage of desorption continuously increased and finally reached 99.6% at 1.0 mol L^−1^ HCl concentration. This result indicates that Ni(II) can be separated with an extremely high performance from MTWB using HCl. Such an adsorbent could be a good choice for industrial applications as it serves high-performance adsorption and desorption capabilities for removing Ni(II) ions from wastewater.

To assess the reusability of MTWB after regeneration, adsorption–desorption experiments were conducted using 0.02 M HCl as the desorption agent. In the first cycle, the adsorbent exhibited nearly complete adsorption and desorption efficiencies, reaching 99.6%. However, in the second cycle, the adsorption capacity sharply decreased to below 30%, indicating a significant loss of performance after the first regeneration. On the other hand, to further evaluate its reuse potential without regeneration, MTWB was subjected to consecutive adsorption cycles using 250 mg L^−1^ Ni(II) ions and 2.0 g L^−1^ of the adsorbent. After three cycles, the adsorption efficiency decreased to approximately 40%. The limited reusability after desorption or without regeneration may be attributed to the irreversible occupation or alteration of active binding sites during the initial adsorption–desorption cycles, as well as possible structural changes in the MTWB matrix induced by acid treatment or heavy metal loading. In conclusion, these results indicate that although MTWB exhibits excellent initial adsorption and desorption performance, its long-term reusability is limited and requires further optimization.

#### 2.2.6. Effect of Co-Existing Ions and Real Wastewater Application

**Figure 10:**
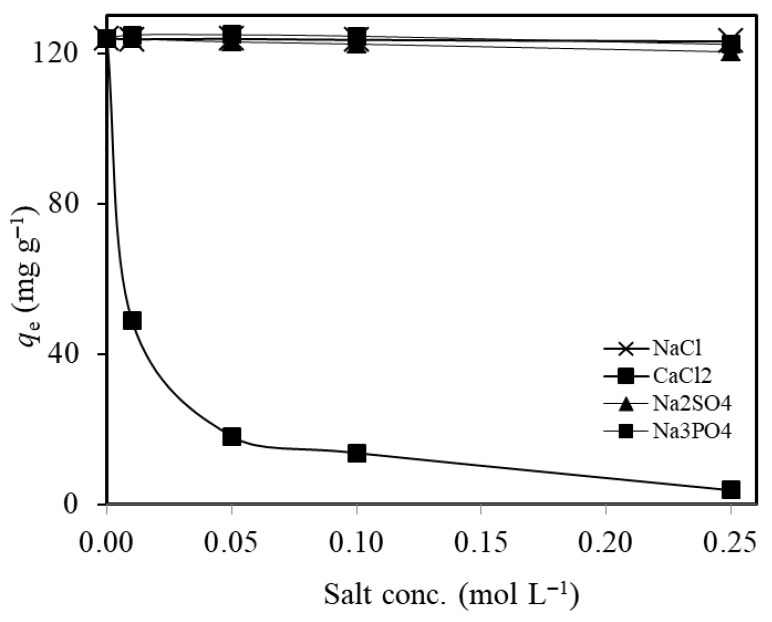
Effect of salt concentration on the adsorption of Ni(II) ions (*C_0_*: 250 mg L^−1^; *V*: 10 mL; initial pH:5.6 (natural); *t*: 15 min; *m_s_*: 2.0 g L^−1^).

A total 10 mL of test solution (pH:5.6), containing 250 mg L^−1^ Ni(II) ions and one of the NaCl, CaCl_2_, Na_2_SO_4_, or Na_3_PO_4_ salts with a definite concentration in 0–0.25 mol L^−1^ range, was added to 2.0 g L^−1^ MTWB and the samples were subjected to adsorption on the mechanical shaker (15 min). Each sample was centrifuged to separate the liquid phase for FAAS analysis. According to the results represented in Figure 10, the Ni(II) adsorption capacity of MTWB is not affected by the presence of NaCl, Na_2_SO_4_, and Na_3_PO_4_. However, the presence of CaCl_2_ inhibits the adsorption of Ni(II) on MTWB. Here, the Ni(II) adsorption capacity of MTWB decreased from 124.0 mg g^−1^ to 3.8 mg g^−1^ as the concentration of CaCl_2_ solution increased from 0 to 0.25 mol L^−1^. Since both Ca^+2^ and Ni^+2^ are divalent cations, competitive adsorption is expected to occur, resulting in a reduction in Ni(II) adsorption capacity. The observed decrease can be attributed to this ionic competition for active binding sites on the adsorbent surface.

To further evaluate the selectivity of the developed MTWB in the presence of co-existing heavy metal ions, additional adsorption experiments were conducted. In these experiments, 100 mg L^−1^ Ni(II) solutions were prepared containing an equal concentration (100 mg L^−1^) of either Pb(II), Cd(II), or Cr(VI), using 2.0 g L^−1^ of MTWB. Additionally, a more complex matrix containing a mixture of 50 mg L^−1^ each of Ni(II), Pb(II), Cd(II), and Cr(VI) was tested under the same conditions. In all cases, Ni(II) removal efficiencies exceeded 95%, demonstrating that the presence of other heavy metals did not interfere with the Ni(II) adsorption performance of MTWB.

To evaluate the applicability of the developed adsorption method for removing Ni(II) ions from real samples with complex matrices, wastewater obtained from a mining company in Gümüşhane Province, Türkiye, was used. The wastewater was spiked with 250 mg L^−1^ Ni(II) ions and 20 mg of MTWB was added. After adjusting the pH to 5.6, the adsorption method was applied under optimum conditions. The results showed a 98% removal efficiency of Ni(II) ions, demonstrating that the developed method can be successfully applied to real samples with highly complex matrices.

#### 2.2.7. ANN Modeling

An artificial neural network (ANN) model was developed using the Neural Network Toolbox (nntool) in MATLAB R2017b to predict the adsorption percentage of Ni(II) ions onto MTWB. Before training, all input and output datasets were normalized to the range [−1, 1] using min-max normalization, as defined by the following equation (Equation (10)):

(10) $$X_{normal}=2\cdot \frac{X_{i}-X_{min}}{X_{max}-X_{min}}-1$$

where *x_i_* represents the actual value, while *x_min_* and *x_max_* denote the minimum and maximum values within the dataset, respectively. Thirty-five experimental datasets were randomly allocated into training (70%), validation (15%), and testing (15%) subsets. The model inputs included initial solution pH, adsorbent dosage (g L^−1^), contact time (min), and initial Ni(II) concentration (mg L^−1^), with adsorption efficiency (%) as the output variable.

**Figure 11:**
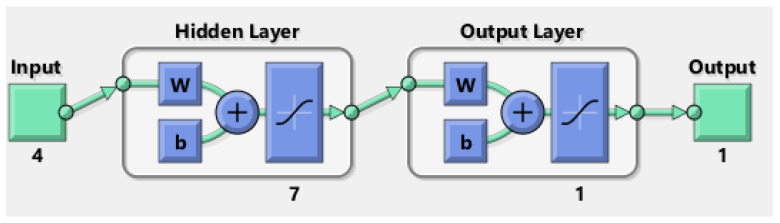
Architecture of the ANN mode.

A feed-forward backpropagation neural network was designed to predict the adsorption efficiency, consisting of an input layer, a single hidden layer, and an output layer. The tangent sigmoid (tansig) activation function was selected for the hidden layer, while the linear (purelin) function for the output layer. Next, the hidden layer architecture was optimized by evaluating neuron counts ranging from 2 to 10. Based on the best predictive performance, the configuration with seven neurons was selected (Figure 11). Network training was conducted using the Levenberg–Marquardt (trainlm) algorithm, which combines the advantages of gradient descent and Gauss–Newton methods, making it particularly suitable for complex nonlinear regression problems due to its fast convergence and high accuracy.

The performance of the developed ANN model was quantitatively assessed using mean squared error (MSE) and the coefficient of determination (*R*^2^) metrics, calculated by the following equations (Equations (11) and (12)), respectively.

(11) $$MSE=\frac{1}{n}\sum_{i=1}^{n}{\left(y_{i}-{\hat{y}}_{i}\right)}^{2}$$

(12) $$R^{2}=1-\frac{{\sum \left(y_{i}-{\hat{y}}_{i}\right)}^{2}}{{\sum \left(y_{i}-{\bar{y}}_{i}\right)}^{2}}$$

where *y_i_* is the observed value, *ŷ_i_* is the predicted value, *ȳ* is the mean of the observed values, and *n* is the total number of samples.

**Figure 12:**
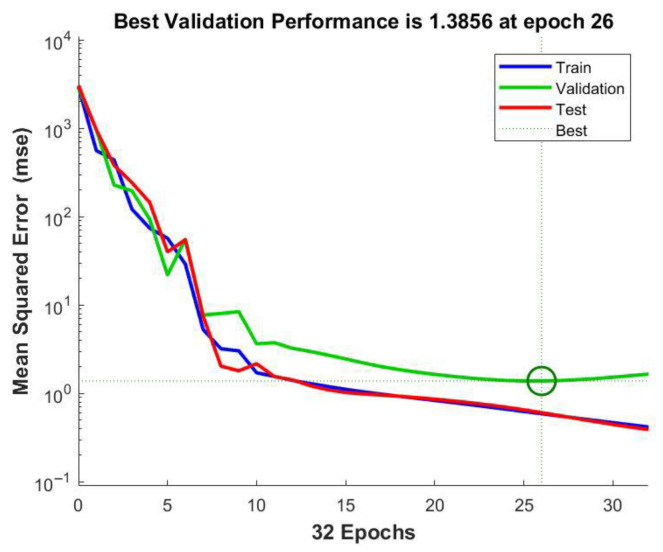
The mean square errors of training, validation, and testing using the Levenberg–Marquardt algorithm.

MSE quantifies prediction accuracy by computing the average squared difference between observed and predicted values; lower MSE values indicate superior model performance (Figure 12).

**Figure 13:**
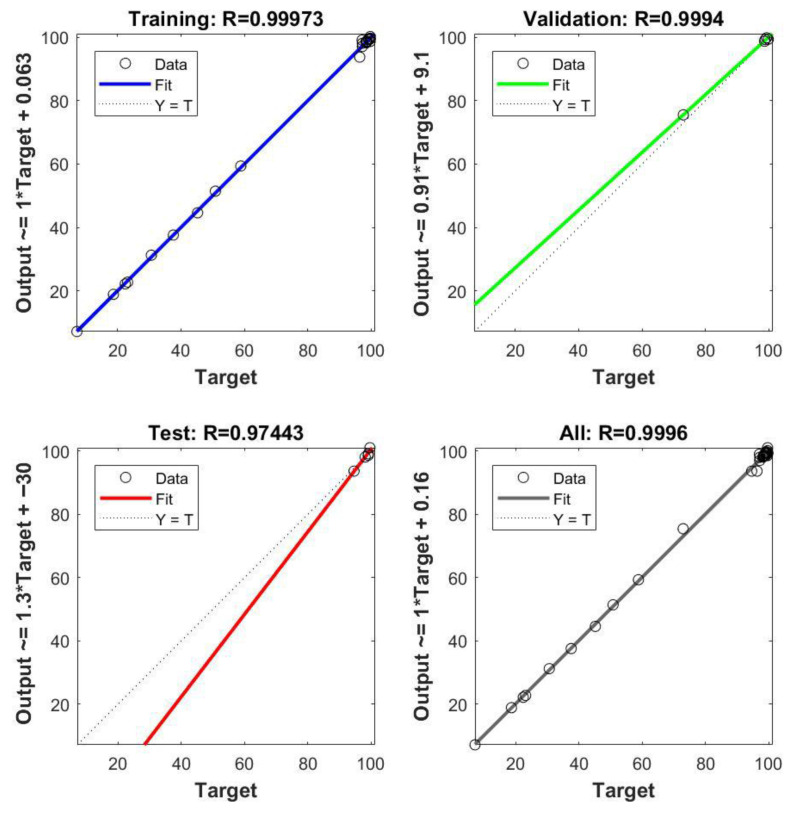
Regression plots of the ANN model for training, validation, testing, and overall datasets.

The *R*^2^ metric measures the proportion of variance in the dependent variable explained by the model, with values approaching 1 indicating strong explanatory power (Figure 13). Both metrics were calculated across training, validation, and test datasets to evaluate the model’s learning efficiency and generalization capability.

As the model predictions were benchmarked against experimental adsorption data using MSE and *R*^2^ as key statistical metrics, the ANN achieved low MSE values of 0.5907, 1.3856, and 0.6069 for the training, validation, and testing subsets, respectively, alongside high *R*^2^ values of 0.9997 (training), 0.9994 (validation), 0.9744 (testing), and 0.9996 (overall) (Figure 13). These results attest to a strong agreement between predicted and observed adsorption efficiencies, confirming that the ANN effectively captures the complex, nonlinear interactions among pH, adsorbent dosage, contact time, and initial concentration of Ni(II) ions. Overall, the developed ANN model proved to be a robust and reliable tool for accurately predicting Ni(II) removal percentages.

## 3. Conclusions

The outcomes of the study clearly demonstrated that modifying tea waste biochar with CuFe_2_O_4_ via the sol–gel method produces a highly efficient and renewable adsorbent for Ni(II) removal. In the study, a waste material that has no usage area but is abundantly available-brewed tea waste, was introduced as a functional support in the synthesis of magnetic composite. The sol–gel method performed during the synthesis stage also provided a homogeneous distribution of magnetic nanoparticles in the biochar matrix, improving structural integrity and functional performance. The adsorption capacity of CuFe_2_O_4_-modified tea waste biochar significantly exceeds most of the values reported for comparable adsorbents, thus introducing a new adsorbent with a very high capacity to the literature. Finally, the successful integration of ANN modeling into the study provides a predictive and scalable tool for future adsorption system design, extending beyond traditional data interpretation. Taken together, these contributions are a step forward in the development of sustainable, high-efficiency adsorbents for environmental remediation.

## 4. Materials and Methods

### 4.1. Chemicals and Apparatus

Copper nitrate trihydrate, Cu(NO_3_)_2_.3H_2_O; iron nitrate nonahydrate, Fe(NO_3_)_3_.9H_2_O; citric acid, C_6_H_8_O; nitric acid, HNO_3_; sodium hydroxide, NaOH; sodium chloride, NaCl; calcium chloride, CaCl_2_; sodium sulfate, Na_2_SO_4_; and sodium phosphate, Na_3_PO_4_ were analytical grade chemicals (obtained from Fluka: Buch, Switzerland, and Merck: Darmstadt, Germany) used in relevant stages of the experimental stages of this study. All solutions were prepared in distilled water. All tests were performed in triplicate.

The Sartorius BP1106 model analytical balance, Hanna p-2221 model pH-meter, IKA RCT Basic model magnetic stirrer, and Santen SE 125 model oven were utilized in the preparation of the materials and solutions. The adsorption was processed on a Edmund Bühler GmbH model mechanical shaker, and then the samples were centrifuged using a BOECO S-8 model device.

The morphology and elemental composition of TWB and MTWB were characterized using a Thermo Scientifıc Apreo 2S scanning electron microscope (SEM), Thermo Scientifıc, Waltham, MA, USA coupled with energy-dispersive X-ray spectroscopy (EDX). The surface functional groups were identified by Fourier transform infrared (FTIR) spectroscopy using a PerkinElmer 1600 FTIR spectrophotometer, PerkinElmer, Springfield, IL, USA. Nitrogen adsorption–desorption isotherms were measured at 77 K using a Micromeritics TriStar II Plus model surface area analyzer, Micromeritics TriStar II Plus, Malvern Panalytical, Worcestershire, UK. Prior to measurement, samples were degassed at 300 °C under vacuum for 3 h. The specific surface area was calculated using the BET method. The total pore volume was estimated from the amount of nitrogen adsorbed at a relative pressure (P/P_o_) of 0.99. The micropore volume was determined using the *t*-plot method. The structural characteristics of TWB and MTWB were analyzed using X-ray diffraction (XRD) measurements performed on a Panalytical X′Pert3 Powder model XRD, Panalytical X′Pert3 Powder, Malvern Panalytical, Worcestershire, UK, with Cu Kα radiation (λ = 1.5406 Å) over a 2θ range of 5° to 80°. The initial and final concentrations of Ni(II) ions were determined using a flame atomic absorption spectrometer (PerkinElmer AAnalyst 400).

### 4.2. Preparation of the Adsorbent Material

#### 4.2.1. Preparation of TWB

The waste, collected as a domestic waste of brewed tea, was filtered through a colander and then left in an open atmosphere for 7 days to remove excess moisture. The material was dried in an oven at 60 °C for 24 h and then transferred to a porcelain crucible to be calcined at 400 °C for 60 min in a muffle furnace with a heating rate of 10 °C/min under oxygen-limited conditions. After cooling down to ambient temperature, the tea waste biochar (TWB) was ground with a power blender to obtain particles smaller than 150 µm in size. This method was a revised version of the one published by Ahmadi et al. (2016) [68].

#### 4.2.2. Sol–Gel Synthesis of CuFe_2_O_4_-Modified Brewed Tea Waste

The method published by Xue et al. (2024) was modified to synthesize copper ferrite using the sol–gel method before the stage of magnetic biochar (MTWB) preparation in this study [69]. A total of 0.05 mol Cu(NO_3_)_2_.3H_2_O and 0.1 mol Fe(NO_3_)_3_.9H_2_O were dissolved in 200 mL of distilled water. The mixture was stirred on a hot plate at 60 °C to obtain a homogeneous solution. Then, 0.2 mol citric acid was added, and the solution was stirred for an additional 15 min. The pH of the solution was adjusted to 7.0–8.0 using diluted NaOH to obtain CuFe_2_O_4_ in a gel media. The chemical reaction is commonly represented as follows [35]:

9Cu(NO_3_)_2 (aq)_ + 18 Fe(NO_3_)_3 (aq)_ + 20 (C_6_H_8_O_7_) _(aq)_ ↓ 9CuFe_2_O_4 (s)_ + 120 CO_2 (g)_ + 80 H_2_O _(l)_ + 36 N_2 (g)_

Then, 10 g of TWB was added to the gel medium and thoroughly mixed on a hot plate at 80 °C for 4 h. After the mixture was dried overnight in an oven at 105 °C, the gel was calcined at 550 °C for 4 h at a heating rate of 10 °C/min under oxygen-limited conditions. As the obtained material cooled down to room temperature in ambient conditions, it was ground and sifted to obtain CuFe_2_O_4_-modified tea waste biochar (MTWB) particles smaller than 150 µm for experimental studies.

### 4.3. Batch Method

MTWB adsorbent was weighed and placed into polypropylene (PP) centrifuge tubes before adding the Ni(II) solution. Batch adsorption experiments scheduled to investigate the effects of parameters such as initial solution pH, contact time, Ni(II) concentration, MTWB dosage, and presence of foreign salts, and batch desorption studies were conducted on a mechanical shaker (300 rpm) using 15 mL PP centrifuge tubes containing test samples of MTWB and Ni(II) ions. Processed samples were centrifuged (3000 rpm) to separate two phases, and the final concentration of Ni(II) in the liquid phase and its initial concentration, both analyzed by FAAS, were used in further calculations (Equation (1)).

### 4.4. Impacts of Parameters and Evaluation of Data

The influences of initial solution pH, contact time, MTWB dosage, and initial concentration of Ni(II) ions, and the presence of foreign salts were parameters investigated to study changes in the adsorption efficiency in this study. Kinetic studies included the evaluation of experimental results using the pseudo-first order, pseudo-second order, and intraparticle diffusion models to define the controlling mechanisms in Ni(II) adsorption onto MTWB. The Langmuir, Freundlich, and Dubinin–Radushkevich isotherm models were used to evaluate data and to characterize the Ni(II) adsorbate–MTWB adsorbent system at equilibrium. An ANN model was developed in MATLAB R2017b (nntool, 9.3.0.713579) to predict Ni(II) adsorption efficiency (%). Input variables included initial solution pH, adsorbent dosage (g L^−1^), contact time (min), and initial Ni(II) concentration (mg L^−1^); the output was adsorption percentage (%). Data were normalized to the range [−1, 1] using min-max scaling and split into training (70%), validation (15%), and testing (15%) sets. The network had one hidden layer with seven neurons (using the tansig activation), and a linear (purelin) output layer. Training was performed using the Levenberg–Marquardt (trainlm) algorithm.

## Figures and Tables

**Table 1:** Kinetic model parameters for the adsorption of Ni(II) ions.

*q_e exp_* (mg g^−1^)	124.5
Pseudo-first order model	
*k*_1_ (min^−1^)	−0.0199
*q_e cal_* (mg g^−1^)	1.81
*R* ^2^	0.8818
Pseudo-second order model	
*k*_2_ (g mg^−1^ min^−1^)	0.071
*q_e cal_* (mg g^−1^)	125.0
*R* ^2^	0.9999
Intraparticle diffusion model	
*k*_id,1_ (mg g^−1^ min^−1/2^)	1.26
*R* ^2^	0.9596
*k*_id,2_ (mg g^−1^ min^−1/2^)	0.14
*R* ^2^	0.9949
*C*	122.2

**Table 2:** Langmuir, Freundlich, and Dubinin–Radushkevich (D-R) isotherm parameters for the adsorption process.

Langmuir isotherm model	
*q_max_* (mg g^−1^)	232.6
*b* (L mg^−1^)	0.125
*R* ^2^	0.9999
Freundlich isotherm model	
*K_f_* (mg g^−1^)	66.8
*n*	5.1
*R* ^2^	0.8457
D-R isotherm model	
*q_m_* (mg g^−1^)	17.7
*β* (mol^2^ kJ^−2^)	−0.002
*E* (kJ mol^−1^)	15.8
*R* ^2^	0.9300

**Table 3:** Table of comparison.

Adsorbent	*q_max_* (mg g^−1^)	Optimum Conditions			Reference
		pH	Dosage	Time	
Banana peel activated carbon	41.53	7	-	60 min	[9]
Acid activated sawmill wood waste products	76.342	5.0	1.2 g/L	100 min	[10]
Unactivated (raw) sawmill wood waste products	68.752	5.0	1.0 g/L	120 min	
Carbon-nanotube-modified biochar (CNT3-CBC800)	32.87	6	1.0 g/L	6 h	[11]
Reduced graphene oxide/bentonite	252.41	10	-	80 min	[12]
Reduced graphe[ne oxide/bentonite/1%ZnO	185	10	-	70 min	
Reduced graphene oxide/bentonite/3%ZnO	125.03	10	-	70 min	
Reduced graphene oxide/bentonite/5%ZnO	96.60	10	-	70 min	
Sugarcane bagasse	53.9	6	-	1 h	[14]
Extracted cellulose	37.0	6	-	1 h	
Carboxymethyl cellulose	152.8	6	-	1 h	
Pineapple leaf biochar	44.88	5.0	2.0 g/L	15 min	[19]
MnFe_2_O_4_ doped hydroxyapatite/kaolinite/biochar	204.680	7.0	2.0 g/L	50 min	[21]
MnFe_2_O_4_ doped hydroxyapatite/vermiculite/biochar	230.340	7.0	2.0 g/L	30 min	
Biochar of Zea mays husk modified by FeSO_4_	22.53	6	-	90 min	[24]
Marine *Chlorella* sp. biochar (ultrasonication adsorption)	27.45	7	0.5 g/L	15 min	[25]
Marine *Chlorella* sp. biochar (conventional adsorption)	24.76	7	0.5 g/L	80 min	
*A. donax* Linn activated carbon	8.614	6.0	4.0 g/L	1 h	[67]
MTWB	232.6	5.6	1.0 g/L	15 min	This study

## Data Availability

The original contributions presented in this study are included in the article. Further inquiries can be directed to the corresponding author.

## Supplementary Materials

The following supporting information can be downloaded at: https://www.mdpi.com/article/10.3390/gels11080628/s1, Figure S1. (a) SEM image of scanned region of TWB and EDX maps of corresponding area for (b) carbon (c) nitrogen (d) oxygen (e) calcium. Figure S2. EDX spectra for TWB. Figure S3. (a) SEM image of scanned region of MTWB and EDX maps of corresponding area for (b) carbon (c) nitrogen (d) oxygen (e) calcium (f) iron (g) copper. Figure S4. EDX spectra for MTWB. Table S1. Percentages of elements in EDX analysis of TWB. Table S2. Percentages of elements in EDX image analysis of MTWB.
