# Supplementary material for: Sol–Gel Synthesized CuFe2O4-Modified Biochar Derived from Tea Waste for Efficient Ni(II) Removal: Adsorption, Regeneration, and ANN Modeling
Source: Gels. 2025 Aug 10;11(8):628. doi: 10.3390/gels11080628 (PMC12385288; doi:10.3390/gels11080628)
Supplement: Supplementary file 1 [file gels-11-00628-s001.zip › gels-3750781-supplementary.pdf]

Article

Sol–Gel Synthesized $CuFe_2O_4$-Modified Biochar Derived from Tea Waste for Efficient Ni(II) Removal: Adsorption, Regeneration, and ANN Modeling

Celal Duran [1,*], Sengul Tugba Ozeken [1], Serdal Seker [2] and Duygu Ozdes [3]

1 Faculty of Sciences, Department of Chemistry, Karadeniz Technical University, Trabzon 61080, Türkiye; stozeken@gmail.com
2 Graduate School of Education, Gumushane University, Gumushane 29100, Türkiye; serdalseker29@yahoo.com
3 Gumushane Vocational School, Gumushane University, Gumushane 29100, Türkiye; duyguozdes@hotmail.com
* Correspondence: cduran@ktu.edu.tr

Table of Contents:

Content	Page
Figure S1	S3
Figure S2	S4
Figure S3	S5
Figure S4	S6
Table S1	S7
Table S2	S7

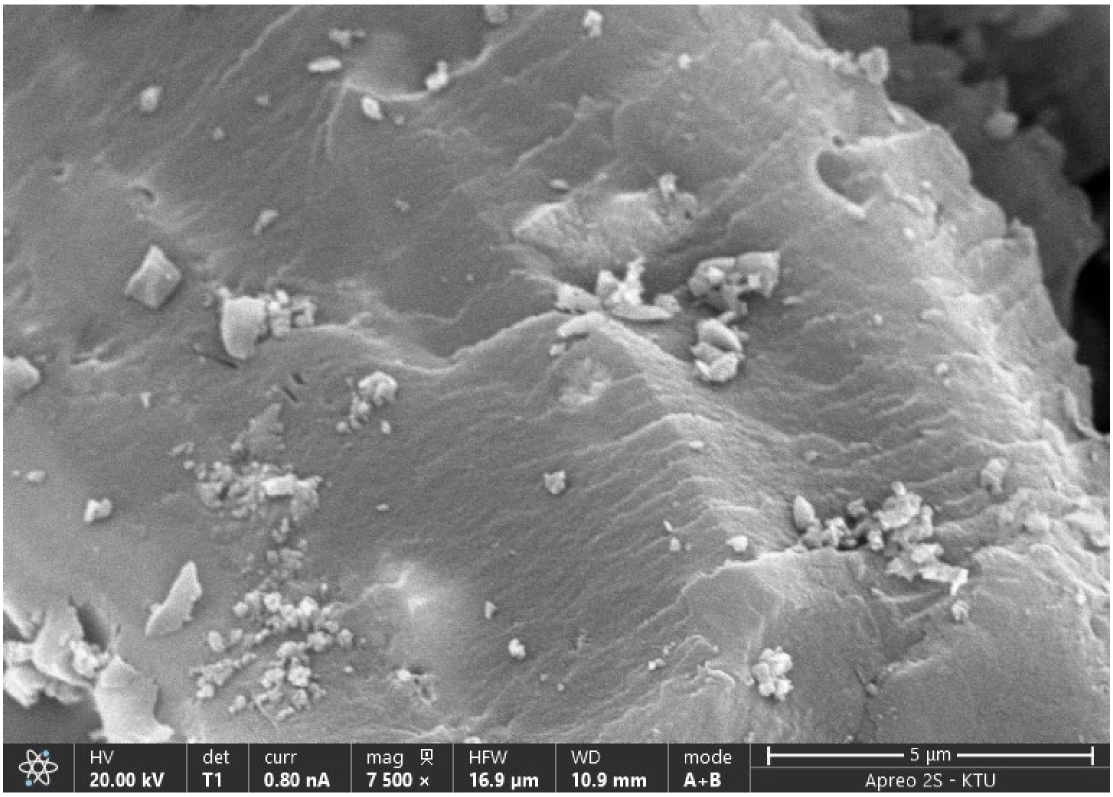

(**a**)

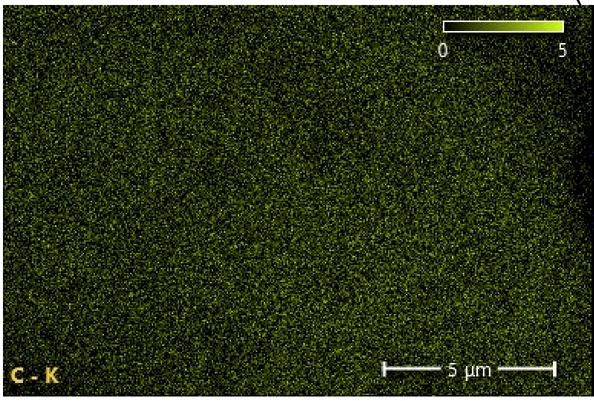

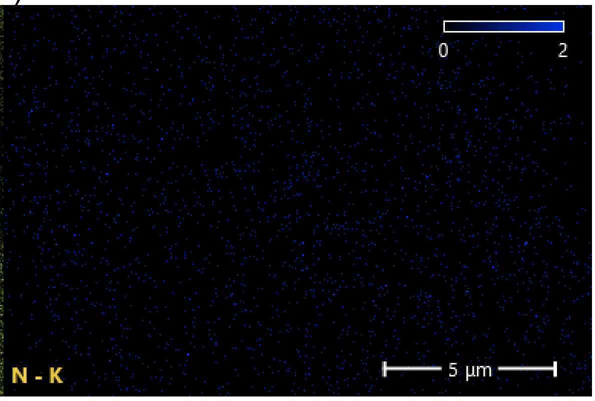

(**b**) (**c**)

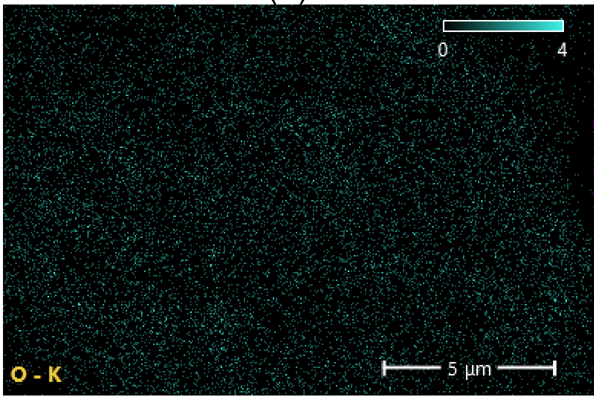

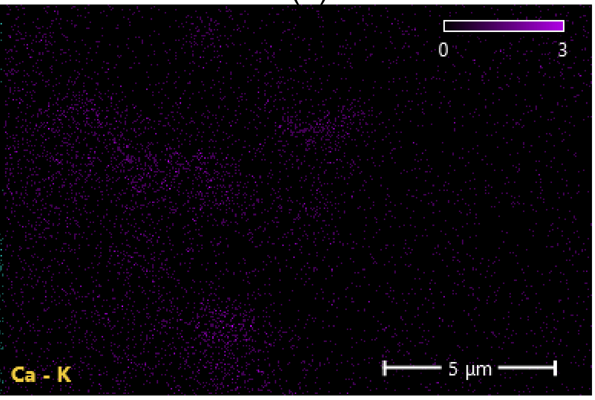

(**d**) (**e**)

Figure S1. (**a**) SEM image of scanned region of TWB and EDX maps of corresponding area for (**b**) carbon (**c**) nitrogen (**d**) oxygen (**e**) calcium.

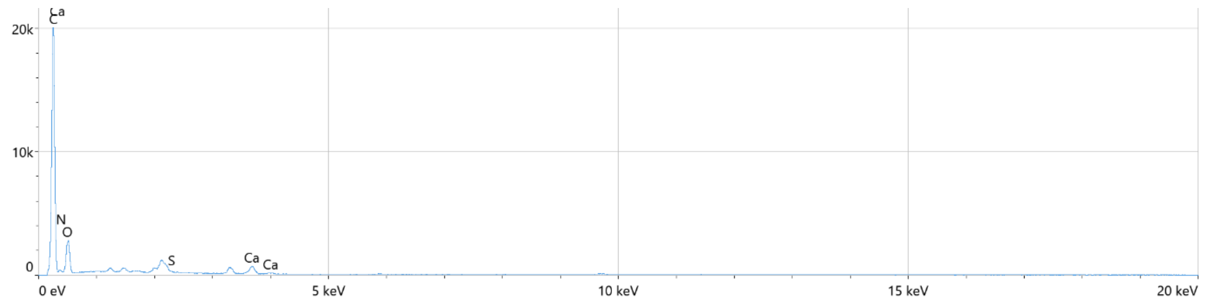

Figure S2. EDX spectra for TWB.

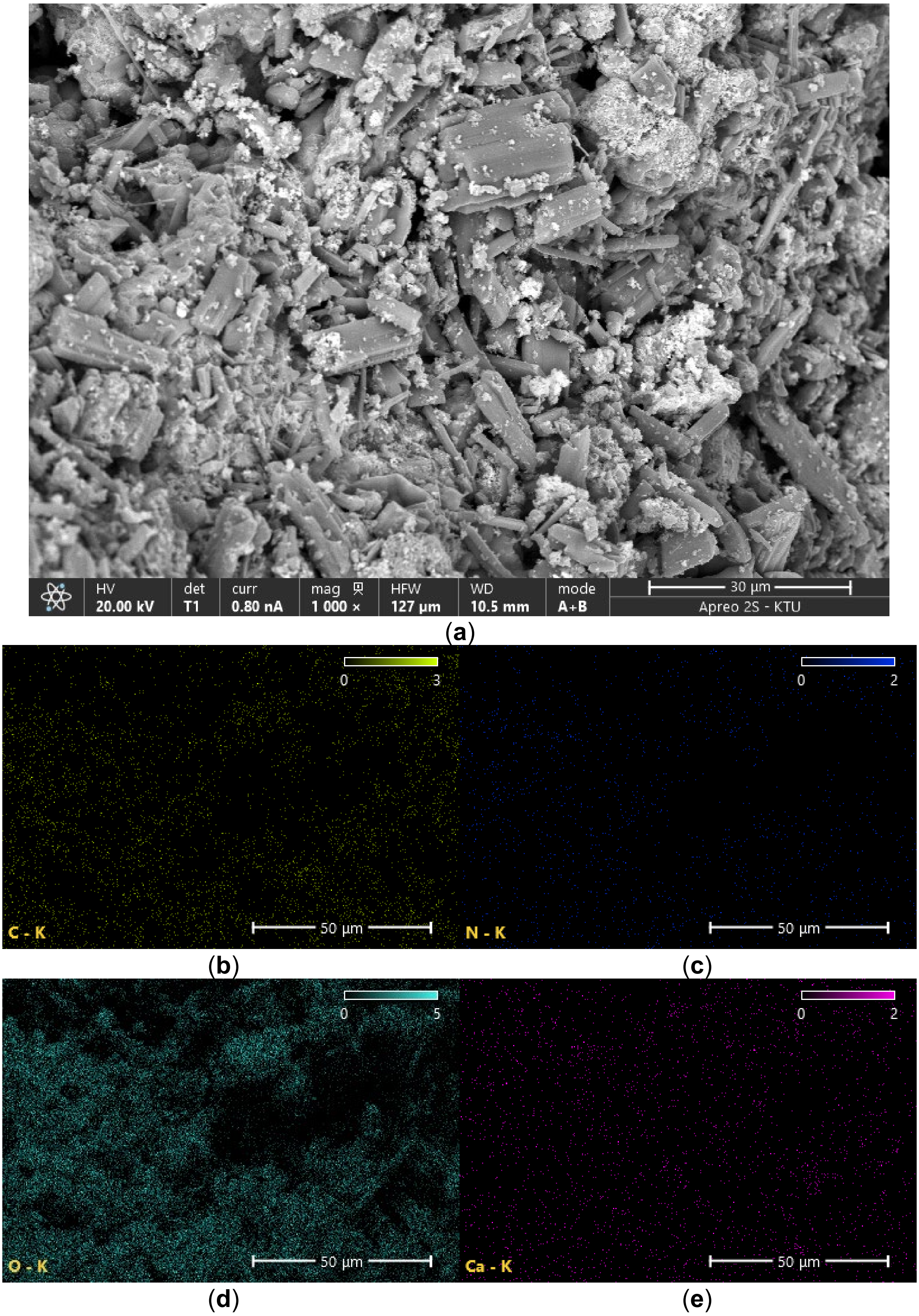
HV
20.00 kV
det
T1
curr
0.80 nA
mag
1 000 ×
HFW
127 µm
WD
10.5 mm
mode
A+B
30 µm
Apreo 2S - KTU
(a)
0
3
C - K
50 µm
(b)
0
2
N - K
50 µm
(c)
0
5
O - K
50 µm
(d)
0
2
Ca - K
50 µm
(e)

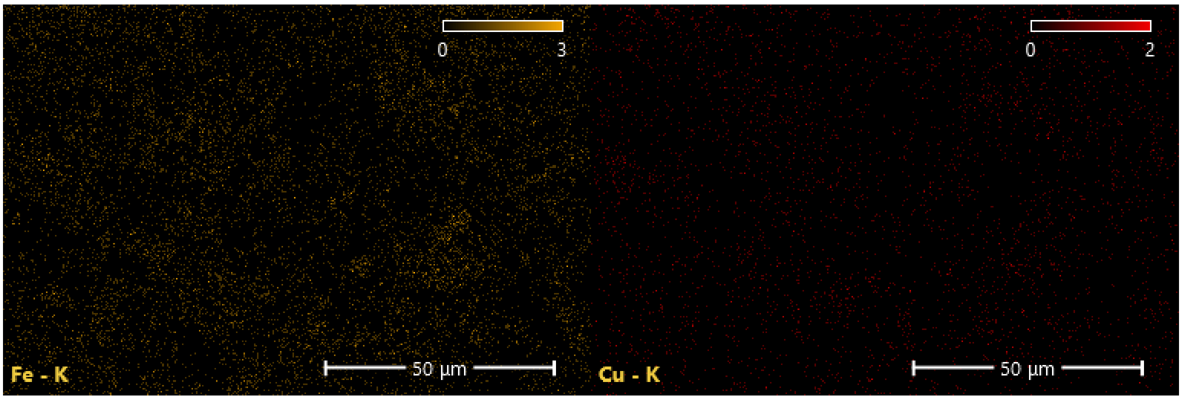

(**f**) (**g**)

Figure S3. (**a**) SEM image of scanned region of MTWB and EDX maps of corresponding area for (**b**) carbon (**c**) nitrogen (**d**) oxygen (**e**) calcium (**f**) iron (**g**) copper.

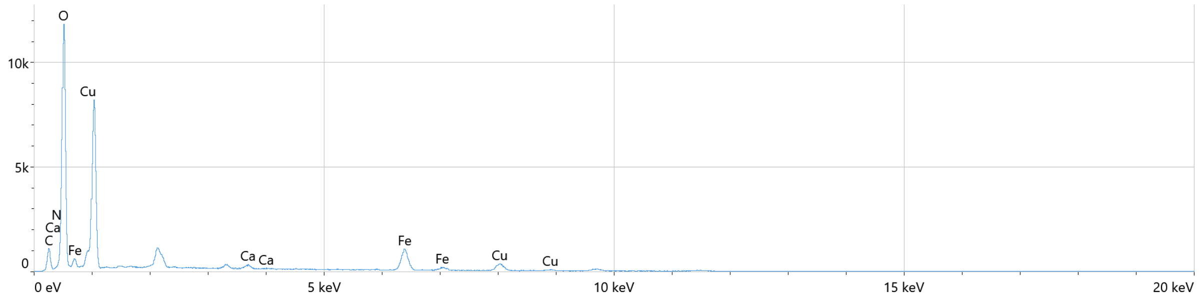

Figure S4. EDX spectra for MTWB.

Table S1. Percentages of elements in EDX analysis of TWB.

Element	Atomic %	Atomic % Error	Weight %	Weight % Error	Net Counts
C	56.3	0.3	49.3	0.3	122 586
N	12.5	1.8	12.7	1.8	2 799
O	30.4	0.5	35.5	0.5	15 909
Ca	0.8	0.0	2.5	0.1	8 317

Table S2. Percentages of elements in EDX image analysis of MTWB.

Element	Atomic %	Atomic % Error	Weight %	Weight % Error	Net Counts
C	13.4	0.3	8.6	0.2	8 198
N	0.5	0.5	0.4	0.4	194
O	78.4	0.5	67.2	0.4	81 794
Ca	0.5	0.0	1.0	0.1	2 625
Fe	4.8	0.1	14.3	0.2	15 986
Cu	2.5	0.1	8.5	0.4	5 342
